# Supplementary material for: Empirical methods for controlling false positives and estimating confidence in ChIP-Seq peaks
Source: BMC Bioinformatics. 2008 Dec 5;9:523. doi: 10.1186/1471-2105-9-523 (PMC2628906; doi:10.1186/1471-2105-9-523)
Supplement: Additional File 1 — A variety of html documents from the USeq web site detailing the available applications, their best usage, output file type descriptions, command line menus, etc. [file 1471-2105-9-523-S1.zip › USeqUserGuides/JavaDoc.zip!/JavaDoc/trans/roc/Sgr.html]

Sgr


|  |  |  |
| --- | --- | --- |
| **Overview** \| **Package** \| **Class** \| **Use** \| **Tree** \| **Deprecated** \| **Index** \| **Help** | |  |
| **PREV CLASS**   **NEXT CLASS** | **FRAMES**    **NO FRAMES**     **All Classes** |  |
| SUMMARY: NESTED \| FIELD \| CONSTR \| METHOD | DETAIL: FIELD \| CONSTR \| METHOD |  |


---


## trans.roc Class Sgr

```
java.lang.Object
  trans.roc.Sgr
```

**All Implemented Interfaces:**: java.io.Serializable

---

``` public class Sgr extends java.lang.Object implements java.io.Serializable ```

Sgr line container.

**See Also:**: Serialized Form

---

**Constructor Summary**

`Sgr(java.lang.String line)`

`Sgr(java.lang.String line,
java.lang.String chromosome)`


| **Method Summary** | |
| --- | --- |
| `java.lang.String` | `getChromosome()` |
| `int` | `getPosition()` |
| `double` | `getScore()` |
| `static java.util.ArrayList<Sgr>` | `loadSgrFile(java.io.File sgrFile)`             Loads an xxx.sgr(.zip) file into an ArrayList of Sgr |
| `void` | `setScore(double score)` |
| `static GrGraph[]` | `splitByChrom(Sgr[] sortedSgrs)`             Splits a Sgr[] by chromosome. |
| `static Sgr[][]` | `splitByChromosome(Sgr[] sortedSgrs)`             Splits a Sgr[] containing many chromosome lines to a Sgr[chrom][lines]. |
| `java.lang.String` | `toString()` |

**Methods inherited from class java.lang.Object**

`equals, getClass, hashCode, notify, notifyAll, wait, wait, wait`

**Constructor Detail**

### Sgr

```
public Sgr(java.lang.String line)
```

---


### Sgr

```
public Sgr(java.lang.String line,
           java.lang.String chromosome)
```


**Method Detail**

### splitByChromosome

```
public static Sgr[][] splitByChromosome(Sgr[] sortedSgrs)
```

:   Splits a Sgr[] containing many chromosome lines to a Sgr[chrom][lines].
    Assumes the Sgr[] is sorted by chromosome!

---


### splitByChrom

```
public static GrGraph[] splitByChrom(Sgr[] sortedSgrs)
```

:   Splits a Sgr[] by chromosome. Assumes the Sgr[] is sorted!

---


### loadSgrFile

```
public static java.util.ArrayList<Sgr> loadSgrFile(java.io.File sgrFile)
```

:   Loads an xxx.sgr(.zip) file into an ArrayList of Sgr

---


### getChromosome

```
public java.lang.String getChromosome()
```

---


### getPosition

```
public int getPosition()
```

---


### getScore

```
public double getScore()
```

---


### toString

```
public java.lang.String toString()
```

:   **Overrides:**: `toString` in class `java.lang.Object`

---


### setScore

```
public void setScore(double score)
```


---


|  |  |  |
| --- | --- | --- |
| **Overview** \| **Package** \| **Class** \| **Use** \| **Tree** \| **Deprecated** \| **Index** \| **Help** | |  |
| **PREV CLASS**   **NEXT CLASS** | **FRAMES**    **NO FRAMES**     **All Classes** |  |
| SUMMARY: NESTED \| FIELD \| CONSTR \| METHOD | DETAIL: FIELD \| CONSTR \| METHOD |  |


---
